# Biomimetic Urban and Architectural Design: Illustrating and Leveraging Relationships between Ecosystem Services

**DOI:** 10.3390/biomimetics6010002

**Published:** 2020-12-30

**Authors:** Maibritt Pedersen Zari

**Affiliations:** School of Architecture, Victoria University of Wellington, Wellington 6011, New Zealand; maibritt.pedersen@vuw.ac.nz

**Keywords:** ecosystem biomimicry, urban ecology, regenerative design, green infrastructure, ecosystem services relationships, ecomimicry, architecture

## Abstract

Redesigning and retrofitting cities so they become complex systems that create ecological and cultural–societal health through the provision of ecosystem services is of critical importance. Although a handful of methodologies and frameworks for considering how to design urban environments so that they provide ecosystem services have been proposed, their use is not widespread. A key barrier to their development has been identified as a lack of ecological knowledge about relationships between ecosystem services, which is then translated into the field of spatial design. In response, this paper examines recently published data concerning synergetic and conflicting relationships between ecosystem services from the field of ecology and then synthesises, translates, and illustrates this information for an architectural and urban design context. The intention of the diagrams created in this research is to enable designers and policy makers to make better decisions about how to effectively increase the provision of various ecosystem services in urban areas without causing unanticipated degradation in others. The results indicate that although targets of ecosystem services can be both spatially and metrically quantifiable while working across different scales, their effectiveness can be increased if relationships between them are considered during design phases of project development.

## 1. Introduction

The way cities and the buildings within them are designed will need to change rapidly and effectively to address converging drivers of change such as climate change and biodiversity loss, while managing human population growth, increased per capita consumption, and urbanisation [1]. Although cities only occupy approximately 3 to 4% of global land area [2,3], they are the sites of tremendous concentrations of energy use, water use, materials, greenhouse gas emissions and other pollutants. Cities are huge drawers and consumers of ecosystem services and producers of waste, but they are also home to large reserves of human ingenuity, labour, economic activity, and cultural wealth. Modern cities are primarily sites for cultural expression and the facilitation of trade, rather than for the production of physical resources or the generation of services that produce tangible physical health, either of ecosystems or humans [4]. Despite this, urban environments must be considered in terms of their impact on ecosystems and their potential role in facilitating regeneration of them. Due to the built environment’s increasing appropriation of the goods and services of ecosystems, vital ecological services for human society (and other species) such as climate regulation, soil formation, nutrient cycling, pollination, and waste assimilation are negatively affected [5]. Several urban design approaches have been recently formulated or expanded in an attempt to integrate performance-based evaluation methods into urban design [6,7]. These methods should be explored for their relevance to regenerative urban design paradigms. However, this paper specifically focuses on emulating ecosystem services as a regenerative urban design strategy. This is a form of urban biomimicry. Urban biomimicry, particularly related to whole-system levels, has been less explored in design disciplines [8] but important concepts and precedents are explored by Pedersen Zari [9], Baumeister et al. [10], Hayes et al. [11] and Taylor Buck [12].

Analysing the urban built environment from the perspective of how ecosystems function (i.e., what they do), and then designing changes to cities so that they begin to emulate the functions of ecosystems (a form of sustainability focused urban biomimicry [9]) could work towards the creation of cities where positive integration with, and restoration of, local ecosystem services could be realised [9,10]. Such a framework of ecosystem services is one way to understand the complexity of ecosystem processes and human interactions with them [13]. If designers and policy makers are to effectively use ecosystem service models and concepts in urban settings, they must understand how these ecosystem services are related [14,15]. This is so potential synergies between ecosystem services can be leveraged, but also so that potential trade-off relationships between certain ecosystem services can be avoided or addressed [16]. To date, little research has examined relationships between ecosystem services for an urban design context (but see: [1,16,17]). This research seeks to address this gap in knowledge. The aim of this research is to define and illustrate key relationships between ecosystem services (where quantitative scientific ecological research exists), so that methodologies that aim to create or enhance urban ecosystem services become more effective, practical, and quantifiable by adding in the ability to plan for, anticipate, and leverage interactions between designed ecosystem services. The intention is that the relationship diagrams presented here become useful tools for research, evaluation of existing built environments, and the design of new developments or retrofits, particularly those seeking to create human and ecological health rather than to simply minimise harm. These diagrams can also form the basis of the future development of interactive design tools [16].

### 1.1. The Importance of Ecosystem Services

Ecosystem services are defined and listed in different ways [18,19,20,21], but typically are divided into provisioning services such as food and medicines; regulation services such as pollination and climate regulation; supporting services such as soil formation and fixation of solar energy, and cultural services such as artistic inspiration and recreation. A focus on ecosystem services has been widely adopted among ecology and policy professionals [21,22], and was formalised by the United Nations’ Millennium Ecosystem Assessment of ecosystems and human wellbeing [20]. In ecology literature, a distinction is often made between ecosystem “services” and ecosystem “functions” [17]. Ecosystem functions are “the habitat, biological or system properties or processes of ecosystems” [23] and are independent of human defined value. Ecosystem services are the benefits that humans derive, either directly or indirectly, from ecosystems that support human physical, psychological and economic wellbeing, and are determined by people’s ability to use them. In this context, ecosystem services are used as a way to understand what it is that ecosystems actually do that is crucial to ongoing life, so that these services may be supported, integrated with, or emulated by the built environment.

There are notable philosophical concerns with defining what ecosystems do as solely for the benefit of humans (for example, the use of the word “services” can imply a utilitarian perspective to ecology) [24], but for the purposes of this research, the ecosystem services framework is a useful way for built environment designers and professionals to understand what their buildings, designed landscapes, urban spaces, and urban infrastructure could be designed to do in addition to more traditional design aims such as: to provide shelter, to be energy efficient, and to have aesthetic value.

Ecosystem services are fundamental to basic human survival and human well-being [20,24]; however, human use of ecosystem services is expanding due to human population increases as well as significant rises in per capita rates of consumption [25]. The global condition of most ecosystem services except for the provisioning of food and raw materials has declined over the past sixty years [26]. Many of these ecosystem services cannot be replaced with current technology [27]. While the negative impacts of the loss of ecosystems and biodiversity (and therefore the services they provide) on people in urban areas are difficult to quantify, there is clear evidence that the loss of urban biodiversity and therefore urban ecosystem services have significant and interrelated negative impacts on:

- Human physical health [28,29,30];
- Human psychological health [31,32];
- Societal and cultural wellbeing [33,34];
- Economic health and stability [35,36].

### 1.2. Ecosystem Services and Urban Environments

The purpose of understanding what ecosystems do from an urban design perspective is to measure past, current, and future environmental performance of the built environment in terms of the provision of ecosystem services so that future spatial and temporal ecology-derived performance goals can be devised [9]. In urban environments themselves, ecosystem services are less well understood [37], but are thought to occur at low rates except for cultural ecosystem services [38]. Despite this, several important urban ecosystem services have been identified and include: air purification, water flow regulation, micro-climate regulation, and carbon sequestration [37]. Typically, these urban ecosystem services come from urban “green spaces” such as forests and parks, or “blue spaces” such as lakes and wetlands and represent important opportunities for novel design interventions, particularly related to increasing resilience to climate change [39,40] along with increasing human wellbeing [41]. Opportunities also exist for green or grey/green hybrid infrastructure and for buildings themselves to produce ecosystem services [9,42,43]. One way to reduce or to reverse the negative impact urban environments have on ecosystems may be to create or re-design urban areas so that they more effectively produce ecosystem services, and therefore reduce pressure on both local and distant ecosystems. Such a strategy works towards several of the United Nations’ Sustainable Development Goals devised in 2015 [44]. Healthier ecosystems more readily provide ecosystem services to humans that cannot be provided by the built environment itself and can enable humans to better adapt to the impacts of climate change [45,46]. This is critical as cities continue to expand and as the climate continues to change [47]. Mimicking what ecosystems do can become the overall ecological performance goal generator for a development, while the specific methods or technologies to achieve the goals can be drawn from a wide range of existing design techniques and tools.

**Figure 1:**
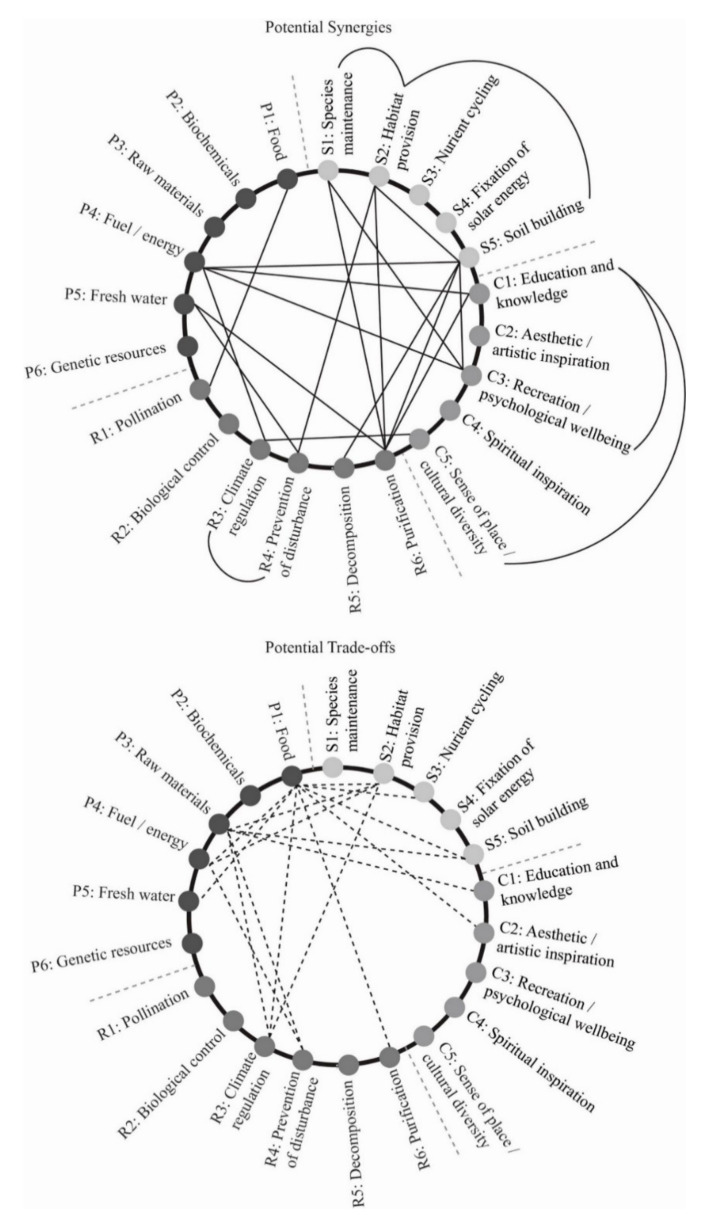
Diagrams of the relationships between ecosystem services (source: [9]).

Just as ecosystems and therefore ecosystem services exist at or across different scales, and are impacted by interventions at different scales, built environment design can be thought of in different nested scales, each with corresponding (and at times overlapping) sets of strategies, techniques, and technologies for influencing ecosystem services. For example, the architectural scale refers to a single building or smaller interventions, the neighbourhood scale is several buildings and the spaces around them, the urban scale is a larger part of a city or the whole city itself, and the regional scale includes urban areas as well as surrounding hinterlands or natural habitat. Scale is undoubtedly an important part of the equation of the relationships between built environment and ecosystem services and should be considered at all design stages. In a similar manner, there is a temporal aspect to design approaches utilising ecosystem services. Timescales, along with the ability of systems to grow and evolve over time, must be incorporated into early stages of design thinking and project development. Understanding relationships between ecosystem services is also important to support the practical application of architectural and urban related ecosystem services design methodologies in [14]. Until Lee and Lautenbach’s [15] work, it was difficult to find ecological literature that defined relationships between ecosystem services (particularly cultural ones) and thus made the concept of applying ecosystem services to a design context less effective and certainly more difficult [1,48]. What is still needed to progress design for urban ecosystem services is graphical illustrations of relationships between urban-focused ecosystem services so that the information is easily and quickly understood by designers and related professionals. The production of Figure 1 aims to address this issue.

### 1.3. Producing Urban Ecosystem Services: Design Approaches

An incremental process that focuses on improving the provision of ecosystem services (not necessarily the original ecosystem itself) to optimal or pre-existing levels in a specific place is a quantifiable starting point in the process of regenerating ecosystem services in the urban built environment. The next stage would be to initiate measures to reintroduce ecosystem services that may be absent in urban areas due to past degradation and removal of ecosystems, or because of conventional ways of constructing buildings or urban environments. This suggests that an urban built environment able to produce more ecosystem services will need to evolve over time rather than be expected to be fully functional after the initial realisation of a design.

While beyond the scope of this paper, understanding, exploring, and employing the parameters and indictors used to measure existing or potential ecosystem services are key to any ecosystem service-based design methodology (see: [9]). Several other combinations of urban parameters may also be useful, particularly when seeking to measure cultural ecosystem services, for example walkability factors [49] or designing for a sense of security [50].

Urban ecological performance targets should be ecologically and culturally specific to a particular site, locality, or region. Systemic approaches that are appropriate to specific places will also vary. This means that the design for urban ecosystem services is highly site-specific. Despite each locality needing to evolve its own unique ecosystem service integration and provision system, knowledge of how to create or begin such systems can be transferred [51]. Designing for increased urban ecosystem services requires design teams to consider which ecosystem services are important or suitable for a particular site before any design of buildings or urban areas begins. Discussions with local peoples and/or ecologists who have knowledge of local ecosystems may further define the hierarchy of importance of the ecosystem services for a specific site and identify an appropriate ecological focus. The following sections outline several methods for developing ecosystem services for architectural and urban design. Several strategies for incorporating ecosystem services into built environment exist as described in the following sections.

#### 1.3.1. Ecosystem Services Analysis and Pre-Development Ecological Baselines

Ecosystems services analysis (ESA) is a means by which the concept of ecosystem services is specifically applied to existing spatial ecological–social contexts (particularly cities). ESA investigates measurable levels of a broader range of ecosystem service provisions that occurred on a particular site when it was an undisturbed ecosystem, and then compares these to the current provision of ecosystem services on the same site (typically now urban) to determine goals for sustainable development that are based on a site’s climatic and ecological reality. This methodology can work at architectural through to urban scales. This methodology is explained in depth by Pedersen Zari [9]. Case studies of three existing cities are provided. The same publication expands upon the details of the ESA process and provides tables of ecological indicators and calculation processes.

Pre-development ecological history analysis was used by the team devising the proposed Lloyd Crossing project for Portland, Oregon. The site’s predevelopment ecosystem functioning was investigated in terms of rainfall, carbon balance, solar energy fixation and habitat type and coverage to determine the ecological performance goals of the project over long time periods [52].

In a similar way, Biomimicry 3.8 (a world leading bio-inspired consultancy offering biological intelligence consulting, and professional training [10]) have developed “Ecological Performance Standards” (EPS) in recent years, which involve investigating an intact ecosystem on or near to a site to quantify ecosystem services, and then derive aspirational performance goals for design based on these [10].

#### 1.3.2. Analogous Ecosystem Methods

The Urban Greenprint project for Seattle, which has grown out of the work of Biomimicry Puget Sound [12], examines the functions of the forest that existed on the site before development. These were studied to determine how buildings and urban spaces on the same site could potentially restore the functions of the predevelopment ecosystems [53]. HOK and Biomimicry 3.8′s Lavasa town development project in India also started from the basis of understanding how the ecosystem on the site had functioned in order to determine development goals [11,54]. In both cases, design ideas were determined by examining organism relationships in existing nearby ecosystems, rather than ecosystem services explicitly.

#### 1.3.3. Interactive Procedural Modelling for Urban Ecosystem Services

Grêt-Regamey et al. [1] described an interactive three-dimensional geographic information system (GIS) based procedural modelling urban design tool with sliding rulers to assist in making ecosystem service trade-offs visually explicit in relation to different urban design scenarios. Their method was tested on a small area of Masdar City in Abu Dhabi and looks at relationships between the ecosystem services of micro-climate regulation, habitat provision, and landscape aesthetics. Results indicate that if relevant spatial variables can be determined, the model could be expanded to incorporate other ecosystem services.

#### 1.3.4. Ecosystem Services Apps and Programmes

Several apps and computer programmes have been developed to enable designers to understand and visualise ecosystem services on specific sites as a basis for design [55,56]. Examples include: Land Utilisation Capability Indicator (LUCI), Artificial Intelligence for Ecosystem Services (ARIES), Integrated Valuation of Ecosystem Services and Tradeoffs (InVEST), Ecosystem Services Identification and Inventory (ESII), and Co$ting Nature. These tools range from complex computer programmes that quantify several ecosystem services over large areas and require a lot of base information and skill to execute (such as LUCI) to more basic apps that can be used on site with limited generic background data to provide approximate quantification and spatial mapping of ecosystem services on site (such as ESII Tool). Usability and outputs of these tools vary greatly, particularly in relation to urban sites, visualisation of data, and suitability of use by designers. Delpy and Pedersen Zari [57] investigated the effectiveness of these tools in different urban contexts.

## 2. Materials and Methods

This research is design-led, rather than a more traditional science-based set of quantifiable experiments. Due to this, this methodology section outlines the steps in the research process and concurrently discusses the reasoning behind each step and how these relate to the research aims, rather than separating these into discrete sections. The methodology used to produce the ecosystem relationship diagrams can be understood as follows:

### 2.1. Step One: Defining Ecosystem Services

A comparative literature-based examination of ecosystem services was conducted in order to define a list of known ecosystem services that relate to an urban context. This research was conducted by the author and is reported on in detail in earlier publications. A summary of this work can be found in [9]. Pedersen Zari [9] provides methodological details regarding the creation of Table 1. The benefit of presenting ecosystem services in a simplified format is that it becomes an easily usable set of generalised ecosystem services that provides an overview for designers with limited background knowledge in ecology. Such a table is of less value to ecologists, where nuances and precise definitions of ecological functions are crucial.

### 2.2. Step Two: Examining Known Relationships between Ecosystem Services

A literature-based review of known ecosystem services associations was conducted. The data used to produce Figure 1 are based on the research of Lee and Lautenbach [15], where 67 field-based case studies reporting on 476 combinations of ecosystem service pairings in the field of ecology were examined and categorised. Further qualifying ecological and socio-economical associations between ecosystem services were provided by Mouchet et al. [58], Howe et al. [59], Bennett et al. [14], and Raudsepp-Hearne et al. [60]. These identified relationships between ecosystem services were then examined to form a series of relationship categories. Haase et al.’s [61] synergy and trade-off matrix was used to understand different types of relationships between ecosystem services including synergistic ones where increased provision of one ecosystem service can result in increased provision of another if managed appropriately, through to trade-off relationships where the provision of one ecosystem service tends to result in the degradation of another.

### 2.3. Step Three: Illustrating Relationships between Ecosystem Services

A design-led research (in this case, graphical analysis) process involving the consolidation and exploration of the results of steps one and two was followed to produce a series of diagrams that illustrate relationships between ecosystem services for a design/architectural/landscape architecture/urban design audience. Diagrams were produced through an iterative design research process. This process culminated in the production of Figure 1.

## 3. Results: Illustrating Relationships between Ecosystem Services for a Design Context

Table 1 presents the results of a comparative review and summary of different ways ecosystem services are discussed in literature (step one of the research methodology). Pedersen Zari [9] provides brief descriptions of what each ecosystem service is in relation to the built environment and which ecosystem services are most suitable for focusing on in an urban design context.

Table 2 shows that new technologies or design methods are not necessarily needed to implement increased ecosystem service provisions in the built environment, and that most of the case studies rely on proven existing technologies or strategies [15]. Rather, there is a need to reconsider the built environment’s overall purpose, and how its performance is evaluated. Emulating what ecosystems do enables design teams to know what the quantifiable ecological goals should be for a development in a specific given location and climate if it is to integrate with existing ecosystems and contribute to their health rather than deplete them. Emulating, rather than just measuring, ecosystem services in urban areas suggests a design strategy based on a systematic transfer of scientific ecological knowledge into a built environment context, rather than design based on simple analogies of ecosystems [62]. In order to know what ecosystems do, it is important to understand and illustrate how ecosystem services are related.

Figure 1 has been prepared as a result of steps 2 and 3 of this research process (see Section 2.2 and Section 2.3). Figure 1 illustrates known synergistic and trade-off relationships between ecosystem services. Where associations are still undecided, have no known effect, or where there is not enough evidence according to ecological literature, associations are not shown. The relationship diagram of ecosystem services (Figure 1) shows that provisioning services are dependent on both regulating and supporting services but supporting or regulating services tends not to be dependent on provisioning services. Due to this, it is important that any ecosystem service-based design methodology or evaluation technique does not ignore regulating or supporting services, although these are more difficult to measure and quantify, and tend to be less well understood by people and therefore perhaps valued [60]. The provision of food for example is the provisioning service that appears to have the most trade-off associations with other ecosystem services (Figure 1). As the ecosystem service of provision of food is fundamental to the continuation of the human species, this reiterates the importance of considering individual ecosystem services in the context of their relationships to others rather than just in isolation. It is physically impossible, or at least very short sighted, to, for example, work towards higher levels of urban food production without also considering climate regulation, soil building, and provision of fresh water at the same time.

This means that design strategies to increase urban ecosystem services should ensure that technologies and systems integrated into a building or wider development are appropriate in terms of overall environmental life cycle impact on multiple ecosystem services. For example, increasing the provision of metals through mining (and thereby increasing the ecosystem service of provision of raw materials) due to some metal’s ability to be recycled many times (and thus engaging more effectively with the ecosystem service of nutrient cycling) could be deemed inappropriate in urban ecosystem service-focused design, given the negative impacts increased mining could have on the ecosystem services of habitat provision and climate regulation as illustrated in Figure 1.

## 4. Discussion: Understanding Relationships between Ecosystem Services for a Design Context

Many examinations of how ecosystems function, or what the services they provide are, result in lists or matrices of unrelated services (see Table 1). Lists are useful in the preliminary stages of design if design teams are unfamiliar with ecosystem services but showing where services are potentially linked (see Figure 1) gives designers insights into how to design buildings, spatial environments, or urban settings to provide or support multiple ecosystem services that potentially positively reinforce each other [73]. Figure 1 also illustrates that when affecting one ecosystem service others need to be considered, so that when improving one, another is not degraded, as is often the case in urban design [1]. For example, increasing the carbon sequestration potential of an area can impact negatively on biodiversity (species maintenance) if both services are not considered in tandem. This could happen for example if trees are planted in an attempt to sequester carbon without an understanding of the tree species necessary in that area to provide habitat for local wildlife.

As the understanding of relationships between ecosystem services and human wellbeing develops, and different values (economic or otherwise) are assigned to different ecosystem services, the relationship diagrams (Figure 1) will need to be refined. Determining the exact levels of causation and representing additional complexities of the relationships would complicate the diagram’s potential as a design tool. Causal patterns are described as being complex, rarely linear and an important part of future ecology research and are needed to progress the understanding of ecosystem services [14,26]. While the relationship diagrams in Figure 1 do not illustrate the complex variety of potential feedbacks and relationships across scales or over time, they do reflect more accurately the physical reality of ecosystem services operating in a dynamic changing context than the simple list presented in Table 1. The development of Figure 1, as results from the field of ecology are made available, should include attempting to illustrate the likely magnitude (both spatial and temporal scales) and reversibility of the synergistic and trade-off relationships as defined by Rodríguez et al. [74].

Indicating where important or obvious relationships exist is useful in a design context because it guides professionals to begin to design interconnected systems rather than to focus on single-issue design goals such as the development of buildings or sites that are just zero waste, carbon neutral, or water positive etc. Such design aims are worthwhile and difficult to achieve, but the point being made is that single focus ecological goals can actually create damage to other ecosystem services if related issues are not understood or considered in tandem [1,17]. The relationship lines in Figure 1 indicate which additional ecosystem services designers or policy makers need to investigate when planning to provide or integrate with a specific ecosystem service. This is crucial both to prevent accidental damage to other ecosystem services through human development where a trade-off relationship is identified, but also to harness strategies for synergistic relationships between ecosystem services.

**Figure 2:**
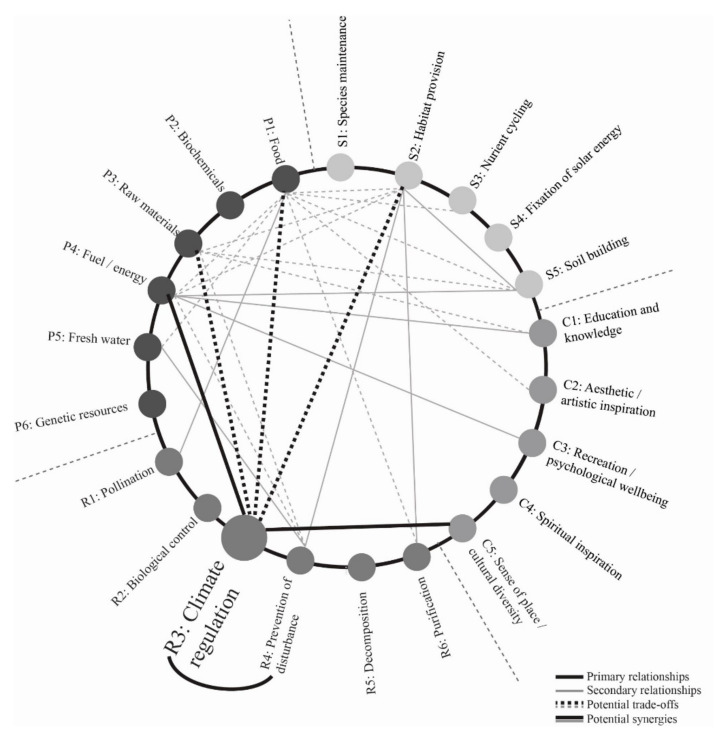
Connections between climate regulation and other ecosystem services. (source: [9]).

Relationship lines between ecosystem services indicated in Figure 1 may also provide pathways to investigate whether certain designs could provide “bundles” of related services. For example, if a design goal for an urban development was to maintain or regenerate climate regulation services, and relationships between ecosystem services are understood and leveraged, the provisioning ecosystem service of energy provision would have to be considered, and the cultural ecosystem service of a sense of place could be enhanced. Careful planning would be needed to ensure no conflicts or reductions in provision of food, raw materials, and habitat ecosystem services. These relationships are illustrated in Figure 2, which demonstrates that just one ecosystem service (climate regulation) is closely related with at least five others. When considering each ecosystem service to which these five are connected, most of the other ecosystem services quickly become additional important considerations. This again highlights the importance of having a holistic and broad approach to designing with or emulating ecosystem services.

When designers combine knowledge of synergetic and trade-off relationships between ecosystem services with specific strategies for increasing certain ecosystem services as detailed in Table 2, strategic decisions can be made about what design potentials/technologies/strategies to explore. For example, returning to the case of climate regulation (Figure 2), a design team working at the architectural (single building) scale may decide to investigate the use of carbon sequestering and/or storing materials for the basic structure, combined with the use of extensive building integrated vegetation to modify local climate and sequester further carbon. By referring to Figure 2, the design team would understand that increasing vegetation in, on, or around a building could serve to not only increase climate regulation ecosystem services (by storing and sequestering carbon), but could also result in a synergistic or “win-win” situation relative to providing multiple ecosystem services with single design interventions, if vegetation is selected not just on carbon sequestration rates, but also relative to habitat provision or food production potentials. Understanding which ecosystem services are related and in which ways helps designers evolve more effective and integrated projects. When combined with a consideration of spatial and temporal scale interactions and impacts, this becomes even more acute.

## 5. Benefits and Challenges of Ecosystem Services Design

Aside from general ecological benefits of built environments that go beyond sustainability and seek to actually create ecological and human health (termed here “ecologically regenerative built environments”), there are significant social and economic benefits such as more resilient communities as the climate continues to change, more equitable communities, potential new revenue streams from buildings, and increased financial value of buildings [75]. Elaboration upon these benefits will not be repeated here, but several additional advantages arise when considering ecosystem services and relationships between them in a sustainable or ecologically regenerative design process. Benefits of incorporating an understanding of ecosystem services into architectural and urban design include increased human health, and increased biodiversity in urban areas [76]. Using ecosystem service research methods to devise design goals enables the success or failure of developments to be gauged from a perspective of site-specific ecological reality. The provision of ecosystem services (past, current and ideal future) can be mapped spatially [17,56,77], and tangible ecological benchmarks for a specific site can be devised over different time periods, lending itself to long term staged urban planning.

When the benefits derived from local ecosystems are understood or become apparent, these are valued more and perhaps therefore preserved [78]. For example, understanding that purer water is a result of nearby forests in a particular city could mean it is easier to convince people of the need to conserve the forest for that purpose (and other ecosystem services) rather than to see and use it as simply a source of timber. This change in thinking has the potential to contribute to prioritising or preventing certain urban development projects in particular areas, and therefore to contribute to effective spatial planning and selection of materials [79]. In addition, by considering impacts on ecosystem services, the implications of urban design and planning decision making can be understood across various spatial boundaries, time scales, and multiple interconnected environmental issues, and can therefore be communicated to citizens, clients, city managers, and other stakeholders. This means more accurate planning and budgeting and perhaps a reduction in a city’s overall ecological footprint.

Several challenges remain before ecosystem services can be more easily and thoroughly integrated into built environment design including ecologists reaching consensus about ecosystem services’ definitions, boundaries, metrics, and hierarchies; defining indicators and benchmarks for measuring the capacity of ecosystems to provide services or recover from damage over time; integrating spatial and temporal issues of both ecosystem services and built environment design as they exist interdependently; exploring how to spatialise, map, model, and visualise ecosystem services in relation to urban environments; categorising existing strategies and methods for producing or integrating ecosystem services in built environment design, and integrating social concerns into ecosystem service methods [16,80,81,82]. It is vital to address these research gaps in order to facilitate the learning and sharing of the concepts, and to enable comparisons to be made between different decisions that have been made. In spite of these challenges and gaps in knowledge, the research described in this article provides a basis for further exploration, experimentation and application of ecosystem service design to urban design situations.

## 6. Conclusions

Cities must become key players in global efforts to conserve and restore biological ecosystems and ecosystem services derived from them, but cities should also begin to produce ecosystem services in higher quantities through the medium of architecture and/or urban blue and green space. If the goal of architecture and urban design is to create or retrofit cities so that they support the wellbeing of people and society, the support and regeneration of urban ecosystem services must be integrated into design decisions and interventions. This may help to reframe the essential human–nature relationship and may be of use to designers or policy makers working to create highly sustainable or even potentially regenerative urban areas. In order to progress this agenda, urban design concepts and methods that enable cities to produce ecosystem services in greater volumes are needed. To make these methodologies more effective, an understanding of how ecosystem services are related is vital.

The concept of ecosystem services is increasingly being applied to many fields of human endeavour, and if extended to architectural and urban design, the potential for profound change in how built environments are designed, valued, built and used is apparent. This is not just related to reducing environmental impacts but also could be part of bringing ecological knowledge into built environment design so that evolving built environments actually begin to contribute positively to ecosystems and produce ecosystem services. The change needed will not necessarily come through devising new technologies, but by the adoption of new mind-sets and goals for how built environments can and should function. Integrating the provision of ecosystem services into architectural and urban design could provide such goals and targets grounded in the physical ecological reality of the planet.

## Figures and Tables

**Table 1:** Ecosystem services with reference to an urban context *(source:* [9]).

1. Provisioning Services (P)	2. Regulating Services (R)	3. Supporting Services (S)	4. Cultural Services (C)
P1 Food -Human (land/fresh water/marine) -Forage P2 Biochemicals -Medicines -Other P3 Raw materials -Timber -Fibre -Stone -Minerals/ores P4 Fuel/energy -Biomass -Solar -Hydro -Other P5 Fresh water -Consumption -Irrigation -Industrial processes P6 Genetic information	R1 Pollination and seed dispersal R2 Biological control -Pest/disease regulation -Invasive species resistance R3 Climate regulation -GHG regulation -UV protection -Moderation of temperature -Moderation of noise R4 Prevention of disturbance and moderation of extremes -Wind/wave/runoff modification -Mitigation of flood/drought/erosion R5 Decomposition -Waste removal R6 Purification -Water/air/soil	S1 Species maintenance -Biodiversity -Natural selection -Self organisation S2 Habitat provision -Habitat for organisms -Reproduction and nursery habitat S3 Nutrient cycling -Regulation of biogeochemical cycles -Retention of nutrients S4 Fixation of solar energy -Primary production/plant growth (above ground, below ground, marine, fresh water) S5 Soil building -Formation -Retention -Renewal of fertility -Quality control	C1 Education and knowledge C2 Aesthetic value and artistic inspiration C3 Recreation, relaxation and psychological wellbeing C4 Spiritual inspiration C5 Creation of a sense of place and relationship -Cultural diversity and history

**Table 2:** Provision of urban ecosystem services with precedents.

Selection of Design Strategies/Technologies to Increase Ecosystem Services			Scale *	Precedents
Provisioning Services	Provision of food & bio-chemicals (P1, P2)	Food growing on public and private land. Development of near-by peri-urban food production belts. Plant based diets. Increased yield renewable food growing techniques. Roof top/façade/interior/vertical food growing. Reduced export of food.	N, U U, R - A-R A R	Nest We Grow, Hokkaido, Japan, 2014. College of Environmental Design UC Berkeley &. Kengo Kuma & Associates [63].
	Raw materials (P3)	Urban agroforestry. Intensive green rooftops. Industrial ecology; design for deconstruction/recycling. Landfill mining.	U A A, N, U U, R	Chartwell School, Seaside (CA), USA. 2007. EHDD Architecture & Taylor Engineering [64].
	Energy/Fuel (P4)	Energy efficiency (behaviour and technologies). Passive solar. Substitution of fossil fuel sources by renewable ones. Building integrated renewable electricity generation.	A A A-R A	Bullitt Centre, Seattle, USA, 2013. Miller Hull [65].
	Fresh water (P5)	Reduction of water demand (behaviour and efficiency). Recycling and treating water on-site. Returning clean waste water to original source if possible. Rain water harvesting; community rain water tanks; collection/production of ‘alternative’ water sources. Water sensitive urban design; green infrastructure. Forest and wetland capture, storage and filtration of water.	A-R A, N A-R A-R A-U U, R	Te Kura Whare for Tūhoe, Tāneatua, New Zealand, 2014. Jasmax [66].
Regulating Services	Pollination (R1)	Increased urban vegetation: green roofs; living walls; urban agriculture; urban forests etc. Planting for increased biodiversity. Built habitat analogues. Pollinator pathways.	A, N, U A-R A, N N, U	Pollinator Pathway Project, Seattle, USA. 2014. S. Bergmann [67].
	Climate regulation (R3)	Carbon sequestration/storage materials and technologies. Regeneration of protected forest. Increased urban vegetation (increased evapotranspiration, shading, and wind buffering). Ecosystem-based adaptation/nature-based solution strategies. Reduced use of fossil fuels through urban planning.	A, N, U U, R U, R A-R N, U	Aldo Leopold Legacy Center, Baraboo (WI), USA. 2007. Kubala Washatko Architects [68].
	Prevention of disturbance (R4)	Ecosystem-based adaptation; nature-based solutions. Grey/green hybrid construction/infrastructure to mitigate flooding and wave/wind damage; stabilise slopes. Urban forest. Wetlands.	A-R U, R U U, R	Chulalongkorn University Centennial Park, Bangkok, Thailand. 2017. by K. Voraakhom [69].
	Purification (R6)	Cessation of pollution of water ways/harbours/aquifers through treatment of storm/grey/black water, and leachates. Cessation of air and soil pollution. Urban forest regeneration and management strategies. Phyto-remediation/bio-remediation; Living Machines; green roofs and facades; green infrastructure; wetlands. Appropriate mechanical plant (air purification / filtration). Pollution remediating/absorbing building materials. Water sensitive urban design; increased porosity.	U N, U U A, N A, N A N, U	Manuel Gea Gonzales Hospital, Mexico City, Mexico. 2014. Elegant Embellishments, Joshua Socolar, WiLaufs & Buro Happold [70].
Supporting Services	Habitat provision (S1, S2, S4, S5, R2, P6)	Preservation of existing forest and vegetation. Careful planning for urbanisation to avoid habitat loss. Provision of measures to counter fragmentation of habitat. Revegetation of green space to provide habitat. Habitat provision on or in buildings. Hybrid grey/green infrastructure strategies.	U, R U, R U, R N, U A, N N, U, R	The Paddock, Castlemaine, Australia. Hes and Biourbem [71].
	Nutrient cycling and decomposition (S3, R5)	Elimination of wastes; separation of waste streams. Cessation of landfilling; landfill mining. Cessation of emission of sewage to ocean. Deconstruction and reuse of materials. Increased use of local or nearby materials. Industrial ecology; ‘Cradle-to-Cradle’ design. Composting and biodegradation.	U, R U, R R A A, N A-U A-U	ReGen Villages (concept). EFFEKT Architects [72].

* Scales: architectural (A), neighbourhood (N), urban (U), regional (R).
